# Longitudinal study of murine microbiota activity and interactions with the host during acute inflammation and recovery

**DOI:** 10.1038/ismej.2013.223

**Published:** 2014-01-09

**Authors:** Clarissa Schwab, David Berry, Isabella Rauch, Ina Rennisch, Julia Ramesmayer, Eva Hainzl, Susanne Heider, Thomas Decker, Lukas Kenner, Mathias Müller, Birgit Strobl, Michael Wagner, Christa Schleper, Alexander Loy, Tim Urich

**Affiliations:** 1 Division of Archaea Biology and Ecogenomics, Department of Ecogenomics and Systems Biology, Faculty of Life Sciences, University of Vienna, Wien, Austria; 2 Division of Microbial Ecology, Department of Microbiology and Ecosystem Science, Faculty of Life Sciences, University of Vienna, Wien, Austria; 3 Department of Microbiology, Immunobiology and Genetics, Max F. Perutz Laboratories, University of Vienna, Wien, Austria; 4 Institute of Animal Breeding and Genetics, University of Veterinary Medicine Vienna, Wien, Austria; 5 Ludwig Boltzmann Institute for Cancer Research, Medical University of Vienna, Wien, Austria

**Keywords:** butyrate, flagellin, metatranscriptomics, mucin, recovery

## Abstract

Although alterations in gut microbiota composition during acute colitis have been repeatedly observed, associated functional changes and the recovery from dysbiosis received little attention. In this study, we investigated structure and function of the gut microbiota during acute inflammation and recovery in a dextran sodium sulfate (DSS)-colitis mouse model using metatranscriptomics, bacterial 16S rRNA gene amplicon sequencing and monitoring of selected host markers. Parallel to an increase of host markers of inflammation during acute colitis, we observed relative abundance shifts and alterations in phylotype composition of the dominant bacterial orders *Clostridiales* and *Bacteroidales*, and an increase of the low abundant *Enterobacteriales, Deferribacterales, Verrucomicrobiales* and *Erysipelotrichales*. During recovery, the microbiota began to resume, but did not reach its original composition until the end of the experiment. Microbial gene expression was more resilient to disturbance, with pre-perturbation-type transcript profiles appearing quickly after acute colitis. The decrease of *Clostridiales* during inflammation correlated with a reduction of transcripts related to butyrate formation, suggesting a disturbance in host-microbe signalling and mucosal nutrient provision. The impact of acute inflammation on the *Clostridiales* was also characterized by a significant downregulation of their flagellin-encoding genes. In contrast, the abundance of members of the *Bacteroidales* increased along with an increase in transcripts related to mucin degradation. We propose that acute inflammation triggered a selective reaction of the immune system against flagella of commensals and temporarily altered murine microbiota composition and functions relevant for the host. Despite changes in specific interactions, the host–microbiota homeostasis revealed a remarkable ability for recovery.

## Introduction

The mammalian intestine is densely populated by billions of bacteria belonging to an estimated 500–1000 different species predominantly classified to the phyla *Firmicutes* and *Bacteroidetes.* Under conditions of health, host and microbiota live in a mutualistic relationship. The host supplies energy sources, and the key roles of the microbiota are the breakdown of nutrients, the synthesis of hormones and vitamins and colonization resistance to pathogens (Nicholson *et al.*, 2012; Stecher *et al.*, 2013). Short chain fatty acids (SCFAs such as butyrate, propionate and acetate) synthesized during metabolism of diet- or host-derived carbohydrates are actively absorbed and contribute to host energy maintenance (Bergman, 1990). Host–microbiota interactions are also important at the immunological level (Saleh and Trinchieri, 2011). Members of the microbiota or their metabolites, for example, *Clostridium* clusters IV and XIV can induce regulatory T-cells, thereby promoting an anti-inflammatory immune response (Atarashi *et al.*, 2011). Butyrate, which is predominantly produced by *Clostridium* clusters IV and XIV, is a main energy source of colonocytes, impacts cell proliferation and differentiation and lowers the risk of colitis and colorectal cancer (Wang *et al.*, 2006; Plöger *et al.*, 2012).

The human intestine has the highest immune activity in the body (Vickery *et al.*, 2011) maintaining homeostasis with commensals but also responding to the invasion of pathogens. In the colon, this homeostasis is guaranteed by the combined action of a mucin layer, which acts as a first physical line of defense, and a tightly regulated network of innate and adaptive immunity. Sensing of microbe-associated molecular patterns activates host-signalling pathways via pattern recognition receptors, including various Toll-like receptors (TLRs). TLR signals activate the synthesis of antimicrobial peptides, maintain tight junctions and trigger cytokine production in intestinal epithelial cells (Kumar *et al.*, 2009).

Disturbances of the intestinal homeostasis caused by external factors (e.g., dietary shifts and antibiotics treatment) can lead to an imbalance in microbiota composition referred to as dysbiosis (Honda and Littman, 2012). Dysbiosis is also associated with inflammatory bowel disease (IBD), a chronic inflammation that is characterized by phases of severe (flare-ups) or no symptoms (remission) (Berry and Reinisch, 2013). Although the exact etiology of IBD remains unclear, several studies suggest that genetic predisposition, environmental factors, an overreacting immune system and the intestinal microbiota contribute to the development of chronic inflammation.

Microbiota alterations frequently observed in chronic colitis are an increase of *Enterobacteriaceae,* shifts in abundance of *Bacteroidetes* and *Firmicutes,* and within-group alterations that can extend to the family level or below (Frank *et al.*, 2007; Berry *et al.*, 2012). Recently, studies applying metagenomics or metaproteomics also investigated functional variations and concordantly reported alterations in metabolic pathways that might affect host–microbiota interactions (Erickson *et al.*, 2012; Morgan *et al.*, 2012). However, investigations on human subjects are mostly limited to the acute phase of inflammation.

With this study we aimed to obtain a more holistic picture of the functional links between host and microbiota during the time course of colitis development and recovery and to investigate microbial functions affected by the inflammation event. We used dextran sodium sulfate (DSS) to induce a reversible form of colitis (Okayasu *et al.*, 1990). Metatranscriptomics was applied to concurrently investigate the active microbial community and its functional properties, and to link functions to bacterial taxa (Berry *et al.*, 2012; Helbling *et al.*, 2012). To investigate abundance shifts of species-level phylotypes, 16S rRNA gene amplicon sequencing was used. Pro- and anti-inflammatory cytokines were monitored as an indicator of the state of the immune system. Finally, correlation networks were generated applying metatranscriptome, amplicon sequence and host inflammation marker data in order to identify associations between host and microbiota.

## Materials and methods

### Animal experiments

Wild-type C57BL/6 mice originating from three litters and 6–8 weeks of age were provided with 2% DSS (molecular weight: 36–50 kDa, MP Biomedicals, Eschwege, Germany) in autoclaved drinking water *ad libitum* for 5 days, after which they received DSS-free drinking water. The DSS dose chosen caused a moderate to severe colitis. Three mice, which received water throughout the time course of the experiment, were used as controls for weight determination. Each sampling day, representatives from at least two litters were killed. The cecum and colon were removed and flushed with phosphate-buffered saline to collect luminal contents (Berry *et al.*, 2012). Flushed lumen contents were homogenized, collected by centrifugation and either snap-frozen for nucleic acid isolation or fixed in 2% paraformaldehyde overnight at 4 °C and then stored in 70% ethanol/30% phosphate-buffered saline at −20 °C until analysis. The intestine was fixed in 2% paraformaldehyde and prepared for fluorescence *in situ* hybridization (FISH), immunohistochemistry and pathology evaluation using established methods (Horino *et al.*, 2008). Hematoxylin and eosin-stained intestinal colon tissue (photomicrographs taken at 100 × magnification) was scored in blind evaluation. For the inflammation score, three subscores (inflammation severity, inflammation extent and crypt damage) ranging from 0–4 were given and added together which could give a maximum possible score of 12. Animal experiments were approved by the institutional ethics committee and conducted in accordance with protocols approved by the Austrian laws (BMWF-66.006/0002-II/10b/2010).

### DNA and RNA purification and preparation of cDNA libraries

Nucleic acid from lumen contents were extracted with a phenol-chloroform bead-beating procedure (Griffiths *et al.*, 2000) and separated into DNA and RNA using the AllPrep DNA/RNA Mini kit (Qiagen, Hilden, Germany), as described before (Berry *et al.*, 2012). DNA and RNA purified from the same extractions were used for 16S rRNA gene amplicon sequencing and cDNA generation, respectively. For double-stranded cDNA synthesis, total RNA was reverse-transcribed using the SuperScript Double-Stranded cDNA Synthesis Kit (Invitrogen, Lofer, Austria) with modifications as described previously (Berry *et al.*, 2012). Single-stranded cDNA was produced from total RNA using SuperScript III reverse transcriptase according to instructions of the supplier (Invitrogen).

### RNA isolation and cDNA synthesis from colon tissue

For RNA preparation from colon tissue, pieces were homogenized in 700 μl RA1 buffer of the NucleoSpin II RNA isolation kit (Macherey–Nagel, Graz, Austria) and processed according to manufacturer's instructions. cDNA was prepared as described previously (Stockinger *et al.*, 2004).

### Metatranscriptome sequencing and data analysis

Double-stranded cDNA libraries were paired-end sequenced using an Illumina HiSeq (Campus Science Support Facilities GmbH, Vienna, Austria). Read pairs were overlapped using FLASH (Magoč and Salzberg, 2011), which yielded between 4.1 and 15.6 Mio reads of ∼170 bp. Metatranscriptomic sequencing data were analyzed following an established double RNA analysis pipeline (Urich *et al.*, 2008). More than 90% of reads were derived from rRNA; the number of putative mRNA reads per sample ranged from 75 305 to 1 166 930 (Supplementary Table S1). Community composition was determined from 100 000 randomly chosen rRNA reads per sample, which were taxonomically assigned using CREST (Lanzén *et al.*, 2012) (bit score=150, top percent=10, minimal support=5). Putative mRNA tags were compared against the NCBI RefSeq database using BlastX and functionally and taxonomically classified using MEGAN and the SEED functional classification scheme therein (bit score=40, top percent=10, minimal support=1) (Mitra *et al.*, 2011). This resulted in 24 190 to 368 950 functionally annotated mRNAs. Metatranscriptomes were generated from three replicates collected on days 1, 5 and 14 and from four replicates collected on days 8 and 25 (Supplementary Table S2). PAST (Hammer *et al.*, 2001) was used for multivariate analysis of metatranscriptome data. Principal component analysis (PCA) was done by eigenvalue decomposition of a data correlation matrix. Unpaired *t*-test and Pearson Moment analysis in SigmaPlot 11 (Systat) were applied for statistical analysis of variance and correlations, respectively.

### 16S rRNA gene amplicon pyrosequencing

Amplicon libraries were obtained using barcoded pyrosequencing primers 909F and 1492R, pooled, purified and quantified as described before (Berry *et al.*, 2012). Pyrosequencing was performed with Titanium reagents on a 454 genome sequencer FLX (Roche, Vienna, Austria). All 20 samples collected on 5 sampling days were subjected to 16S rRNA amplicon sequencing, but one sample from day 1 was excluded from analysis due to low sequencing depth. Reads were quality-filtered using the amplicon pipeline of the GS Run Processor (Roche) and the Pyronoise algorithm in mothur (Schloss *et al.*, 2011). Operational taxonomic units were generated at 97% sequence identity using UCLUST, as described (Berry *et al.*, 2012). Amplicon sequencing libraries, which had a mean number of 17 999 reads (range [4415–26 495]) of an average length of 269 nt were re-sampled at 3500 reads for α- and β-diversity analyses using QIIME (Caporaso *et al.*, 2010).

### FISH analysis

FISH was performed on paraformaldehyde-fixed biomass, as previously described (Berry *et al.*, 2012). Probes specific for *Bacteroides acidifaciens* (OTU 1812) and *Bacteroidales* OTU 183 were designed based on near full-length 16S rRNA genes recovered from screening of clone libraries established with primer pair 8F and 1492R (Lane, 1991). Probe specificity was evaluated by checking the probes against the SILVA SSU NR108 database (Quast *et al.*, 2013) and stringency of hybridization conditions was optimized using either clone-FISH (Schramm *et al.*, 2002) or environmental samples containing abundant target populations. For *Bacteroides acidifaciens* (OTU 1812) probes Bacid87 I (S-S-Bac-0087-a-A-24, 5′-GCGCCGGTCGCCATCAAAAGTTTG-3′) and Bacid87 II (S-S-Bac-0087-a-A-22, 5′-GCCGGTCGCCATCGGAAGTTTG-3′) were used with 35% formamide. For *Bacteroidales* OTU 183, probe Bada183_437 (S-S-Bada-0437-a-A-22, 5′-CGCCCTTTGCTCCCTGACAAAA-3′) was used with 30% formamide. Probes were deposited at probeBase (Loy *et al.*, 2003).

### Quantification of selected genes and transcripts by qPCR

Copies of 16S rRNA genes from selected taxa and selected microbial transcripts were quantified by qPCR using a Mastercycler ep realplex (Eppendorf, Vienna, Austria). Reaction mixtures (20 μl) contained 10 μl QuantiFast SybrGreen (Qiagen), 1 μl of each of the specific primers (final concentration 0.25 μM, Supplementary Table S2) and 1 μl of template. Running conditions were 95 °C for 5 min, followed by 40 cycles of 95 °C for 10 s, annealing for 15 s with the temperature specified in Supplementary Table S2, and 72 °C for 30 s followed by melting curve analysis. Standard curves were generated as described before (Berry *et al.*, 2012). Gene and transcript copy numbers were calculated per μg DNA or RNA, respectively. Flagellin expression levels were calculated as the ratio of transcript copies and gene copies per μl nucleic acid preparation. Unpaired *t*-test and one-way ANOVA combined with an all pairwise multiple comparison procedure (Student–Newman–Keuls Method) were applied for statistical analysis of variance (SigmaPlot 11).

For mouse gene expression analysis, qPCRs were run with 60 °C annealing temperature on an Eppendorf realplex cycler in a final volume of 15 μl containing 1.5 μl cDNA and 0.3 μM primer using GoTaq qPCR Master Mix (Promega, Mannheim, Germany). Primers used are summarized in Supplementary Table S3. Significance between control (day 1) and test conditions (days 5, 8, 14 and 25) was calculated using the Relative Expression Software Tool (Pfaffl *et al.*, 2002) software via a bootstrapping approach (Pairwise Fixed Reallocation Randomisation Test). 2000 random reallocations of samples were performed; glyceraldehyde 3-phosphate dehydrogenase was employed as a house-keeping gene; comparable results were obtained using hypoxanthine-guanine phosphoribosyltransferase (data not shown).

### Correlation networks

To construct correlation networks, qPCR data from host markers, OTU relative abundances from 16S rRNA gene amplicon pyrosequencing and the relative abundance of transcripts from SEED categories level 1 from metatranscriptome libraries were combined. For sequencing-based data, any OTU or transcript class that did not reach at least 1% relative abundance in any sample was excluded from the analysis. Data was log-normalized and the Pearson correlation coefficient was generated. Pearson correlation coefficients *r*>0.6 or *r*<−0.6 were used to construct the correlation networks in R software environment, which were then visualized using Cytoscape (Shannon *et al.*, 2003).

### Data access

Amplicon and metatranscriptomic data were archived at NCBI Sequence Read Archive under SRP008057 and SRX314796, respectively.

## Results

### Host markers of acute colitis

**Figure 1:**
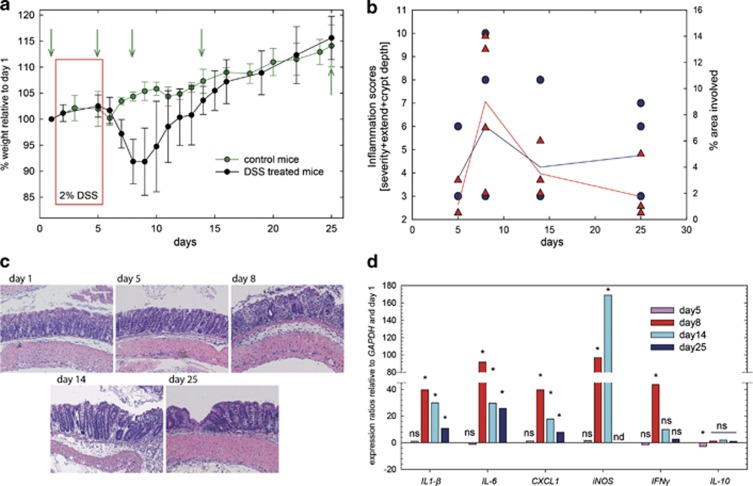
Host parameters during colitis and recovery. (**a**) Weight curves of untreated (control) and DSS-treated mice. Colitis was triggered by the addition of DSS (2%) to drinking water for 5 days (red box); control mice received un-amended drinking water during the course of the experiment. Samples were taken before (day 1) and after DSS treatment (day 5), during acute colitis (day 8) and recovery (days 14 and 25) (green arrows). (**b**) Inflammation scores (calculated as the sum of inflammation severity and extend, and crypt depth) and area of inflammation (%). (**c**) Representative photomicrographs (100 × magnification) of hematoxylin and eosin-stained colon tissues obtained from each sampling date. (**d**) Expression ratios of pro- (*IL-6*, *IL1-β*, *IFNγ* and *CXCL1*) and anti- (*IL-10*) inflammatory cytokines and chemokines and markers of inflammation (*iNOS*) calculated relative to expression at day 1 and *GAPDH*. Expression ratios were calculated using REST, asterisks (*) indicate significance of expression ratios as determined using Pair Wise Fixed Reallocation Randomisation Test (Pfaffl *et al.*, 2002). NS, not significant; ND, not detectable.

Development and remission of DSS-induced colitis in mice was determined by weight measurements and histology scores, and by quantification of expression of pro- (*IL-1β*, *IL-6* and *IFNγ*) and anti-inflammatory (*IL-10*) cytokines, the chemokine *CXCL1* and the inducible nitric oxide synthase. DSS-treated mice started to lose weight at day 5 and reached their lowest weight at days 8 and 9 (Figure 1a). At day 14, weight had returned to day 1 levels, while at day 25 mice had gained 15% compared to their starting weight. This weight gain was comparable to the weight gain of control mice not receiving DSS (Figure 1a). Tissue damage peaked at day 8 and was slightly decreased at day 14; the proportion of affected area declined even further to day 25 (Figures 1b and c). *IL-10* was significantly downregulated only at day 5; *IL1-β*, *IL-6* and *CXCL1* were upregulated (*P*<0.05) at day 8 and remained at significantly higher transcriptional level at days 14 and 25 (Figure 1d). *IFNγ* and inducible nitric oxide synthase were significantly higher expressed at days 8 and 14 (inducible nitric oxide synthase only), but not detectable or not significantly different at day 25, indicating resolution of acute inflammation (Figure 1c).

### Temporal changes in microbiota composition

**Figure 2:**
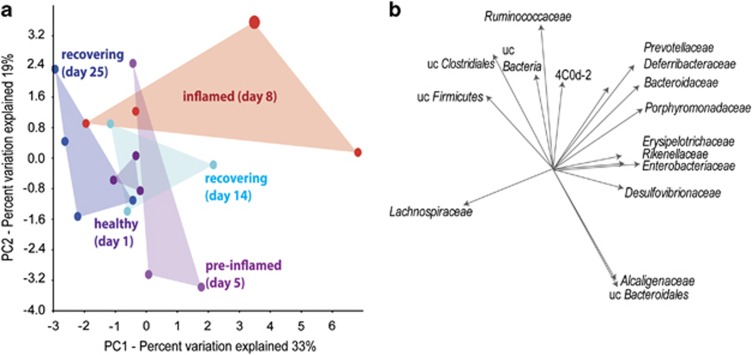
Microbiota dynamics during acute colitis and recovery. (**a**) Score and (**b**) loading plot indicating the correlation between bacterial communities and the health state of the animals. PCA plots were calculated based on relative abundance of metatranscriptomic 16S rRNA reads of bacterial families. The score plot shows the relationship of individual samples. The loading plot defines the contribution of the original variables (bacterial families) to PC1 and PC2 but also indicates the relationship variables to each other, which were further determined by Pearson moment correlation analysis. uc, unclassified, 4C0d-2 *Cyanobacteria-*like lineage.

16S rRNA transcripts of the metatranscriptomes were used to investigate temporal alteration in microbial community composition. The bacterial phyla *Firmicutes* and *Bacteroidetes* contributed the majority of 16S rRNA transcripts (together 96–97%); with *Clostridiales* and *Bacteroidales* being the two dominant orders (together 94–95%, Supplementary Figure S1A). We applied the 16S rRNA transcripts as a measure of relative abundance of bacterial groups even though the rRNA content does not necessarily reflect cellular abundance and is affected in complex ways by the physiological state of the cell. PCA of the relative abundance of bacterial groups from 16S rRNA transcripts revealed that microbiota shifts appeared at the end of DSS treatment (day 5) and further alterations occurred at the peak of inflammation (day 8) (Figure 2a). At day 14, the microbial community began to resemble the community before inflammation, but did not reach its original composition even by day 25. PCA furthermore indicated a higher degree of community variability between replicates in the inflammation and recovery phases (Figure 2a). Shifts from the healthy to an inflamed and recovering microbiome occurred primarily along the first principal component; the clostridial family *Lachnospiraceae* was associated with the healthy status, while members of the *Bacteroidetes* (*Bacteroidaceae, Prevotellaceae, Porphyrmonadaceae* and *Rikenellaceae*) were indicative of inflammation (Figure 2b). The increase of *Bacteroidetes* 16S rRNA occurred in two stages: unclassified *Bacteroidales* peaked at day 5, while members of the *Bacteroidaceae, Porphyrmonadaceae* and *Prevotellaceae* were most abundant at day 8 (Supplementary Figure S1C). Some bacterial families low in relative 16S rRNA abundance, like the *Enterobacteriaceae, Verrucomicrobiaceae* (mainly *Akkermansia* spp.)*, Erysipelotrichaceae* and *Deferribacteracae* (mainly *Mucispirillum* spp.), also increased during acute inflammation, but 16S rRNA reads of these families returned to abundance levels of healthy mice at day 14, with the exception of the *Erysipelotrichaceae* (Supplementary Figure S1D), which only decreased by day 25. Generally, shifts in relative abundance of 16S rRNA of *Clostridiales* and *Bacteroidales* and the increase of *Deferribacteraceae* and *Enterobacteriaceae* 16S rRNA were most pronounced in mice that suffered from greatest weight loss and the most severe inflammation (Supplementary Table S4). During recovery, the ratio of *Clostridiales* to *Bacteroidales* 16S rRNA significantly increased compared to day 1 (Supplementary Figure S1B).

In a previous study (Berry *et al.*, 2012), we showed that many microbiota rearrangements during acute colitis are hidden when 16S rRNA (gene) sequences are grouped on phylum, order or family level. To also determine composition shifts of roughly species-level phylotypes, which is not possible with rRNA reads derived from shotgun metatranscriptomes, 16S rRNA gene sequences derived from amplicon pyrosequencing were analysed. Similar to the metatranscriptomic data, principal coordinates analysis based on weighted and unweighted Unifrac distance indicated a health-state dependent clustering of bacterial communities (Supplementary Figure S2A–D). Phylotype richness had already decreased before the onset of inflammation (day 5) and only started to recover at day 14, whereas community evenness was generally not affected (Supplementary Figure S3).

To investigate compositional dissimilarity within bacterial groups across sampling days, Bray–Curtis dissimilarity index was calculated based on relative abundances or presence and absence of phylotypes (Supplementary Figure S4). In general, a shuffling of abundances rather than a replacement of community members was the cause for the observed community shifts. Shifts were more pronounced for members of the *Bacteroidetes* (*Bacteroidales,* unclassified *Bacteroidales* and *Bacteroidaceae*) than for the *Firmicutes*, and appeared already at day 5. Bray–Curtis dissimilarity based on relative abundance of phylotypes assigned to *Bacteroidaceae* and *Lachnospiraceae* varied to a greater extent at days 14 and 25 than in healthy mice (day 1).

### Bacterial gene expression during colitis and recovery

**Figure 3:**
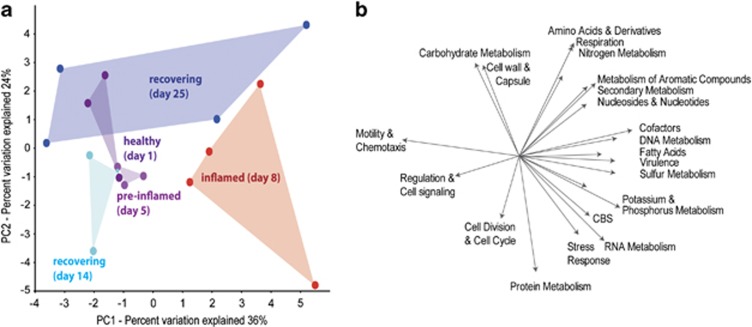
Shifts in microbiota gene expression during acute colitis and recovery. (**a**) Score and (**b**) loading plot indicating the correlation between bacterial mRNA expression and the health state of the animals. PCA plots were calculated using relative abundance mRNA transcript data of level 1 SEED categories. The score plot shows the clustering of samples obtained on different treatment days. The loading plot defines the contribution of the original variables (SEED categories) to the principal components and indicates the relationship variables to each other, which was further determined by Pearson moment correlation analysis. CBS, SEED category ‘Clustering-based subsystems' Cofactors, ‘Cofactors, Cofactors, Vitamins, Prosthetic Groups, Pigments'.

In healthy mice, 69–70% of all putative bacterial mRNAs were assigned to *Firmicutes* (44–47%) and *Bacteroidetes* (23–26%) (53–56% to *Clostridiales* and *Bacteroidales*). ‘Protein Metabolism' and ‘Carbohydrate Metabolism' were the most abundant SEED categories; ‘Virulence' and ‘Motility and Chemotaxis' were the third major categories in *Bacteroidetes* and *Firmicutes* metatranscriptomes, respectively (Supplementary Figures S5–7). PCA generally clustered mRNA profiles according to the day of treatment (Figure 3a). Early changes in gene expression were already observed at day 5 but became most extensive on day 8 (Figure 3a). mRNA reads assigned to the categories ‘Motility and Chemotaxis' and ‘Regulation and Cell Signalling' were associated with healthy and recovering microbiomes (Figure 3b) and the former category correlated positively with *Clostridiales* and *Lachnospiracae* (*r*: 0.516 and 0.528, *P*<0.05). Associated with inflammation were transcripts of ‘Virulence', ‘Sulfur Metabolism', ‘Stress Response' and ‘RNA Metabolism' (Figure 3b), all of which positively correlated with the *Bacteroidales* (*r*: 0.522, *r*: 0.625, *r*: 0.555 and *r*: 0.498, respectively, all *P*<0.05,). During acute colitis, transcripts of ‘Motility and Chemotaxis' were significantly reduced compared to day 1, while relative abundances of mRNA reads affiliated with ‘RNA Metabolism', ‘Clustering-based subsystems', ‘DNA Metabolism', ‘Cofactors, Vitamins, Prosthetic Groups, Pigments' and ‘Fatty Acids' were significantly increased (Supplementary Figure S5).

### Acute colitis is associated with decreased expression of butyrate synthesis genes

**Figure 4:**
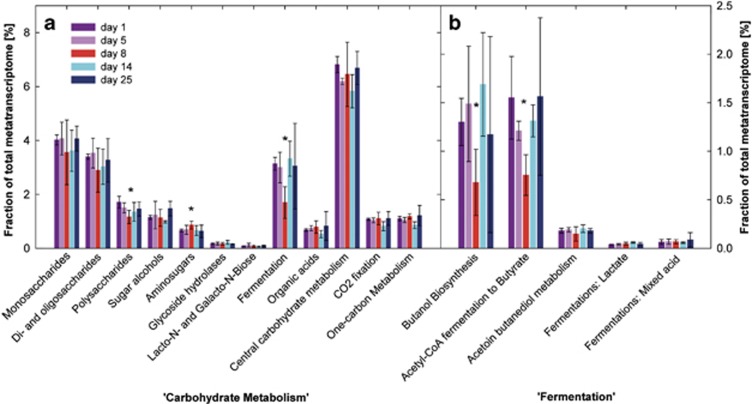
Shifts in ‘Carbohydrate metabolism'-related transcripts. Shown are relative abundances of transcripts of the categories (**a**) ‘Carbohydrate metabolism' and (**b**) ‘Fermentation' assigned with MEGAN at days 1, 5, 8, 14 and 25 in relation to all mRNA reads. Means at days 1 and 8 were compared with unpaired *t-*test, *P*<0.05 was considered significantly different (*).

As carbohydrates are major energy sources of the gut microbiota, we more closely analysed transcripts of the category ‘Carbohydrate Metabolism', which contributed ∼20–25% of all transcripts to the metatranscriptome (Supplementary Figure S5). The relatively high abundance of transcripts assigned to ‘Fermentation‘ and its subcategory ‘Acetyl-CoA-fermentation-to-butyrate' and ‘Organic acids' (subcategory ‘Propionyl-CoA to Succinyl-CoA Module') reflected the major fermentative pathways of *Clostridiales* and *Bacteroidales,* that is, butyrate production and succinate-propionate conversion (Macy *et al.*, 1978; Pryde *et al.*, 2006), respectively (Supplementary Figure S8). Yet, whereas the relative abundance of transcripts related to succinate-propionate conversion remained unaffected, those associated with butyrate formation were significantly reduced on day 8 (Figure 4, Supplementary Figure S8). This decrease occurred within the entire metatranscriptome as well as within the metatranscriptome of *Firmicutes* (Figure 4, Supplementary Figure S8). The key enzymes acetyl-CoA acetyltransferase, which converts acetyl-CoA to acetoacetyl-CoA, 3-hydroxybutyryl-CoA dehydratase catalyzing the dehydration of 3-hydroxybutyryl-CoA to crotonyl-CoA and butyryl-CoA dehydrogenase converting acetacetyl-CoA to butyryl-CoA, were significantly reduced on day 8 compared to day 1 (Supplementary Table S5). *Clostridiales* possess two pathways for the final conversion of butyryl-CoA to butyrate (Louis and Flint, 2009). Butyrate kinase and phosphate butyryltransferase, which form butyrate from butyryl-CoA via the intermediate butyryl-phosphate, were not affected by acute colitis (Supplementary Table S5). Transcripts of butyryl-CoA:acetate CoA-transferase (ButCoA transferase) transfering the CoA of butyryl-CoA to external acetate were not detected within the metatranscriptomes. However, a significant decrease could be determined by quantitative PCR (day 1: log4.58±0.59, day 8: log3.06±0.35, transcripts μg^−1^ RNA). In confirmation, a decrease of both ButCoA transferase transcripts and genes was also observed in independent additional experiments (Supplementary Figure S9). Additionally, transcripts associated with the category ‘Butanol Biosynthesis' were significantly lower on day 8 (Figure 4). This category was mainly represented by pyruvate-formate lyase transcripts of *Firmicutes.* Also significantly reduced was ‘Polysaccharide Metabolism' due to decreased contribution of *Bacteroidetes* transcripts (Figure 4, Supplementary Figure S8).

### Decrease of clostridial flagellin transcripts and genes

**Figure 5:**
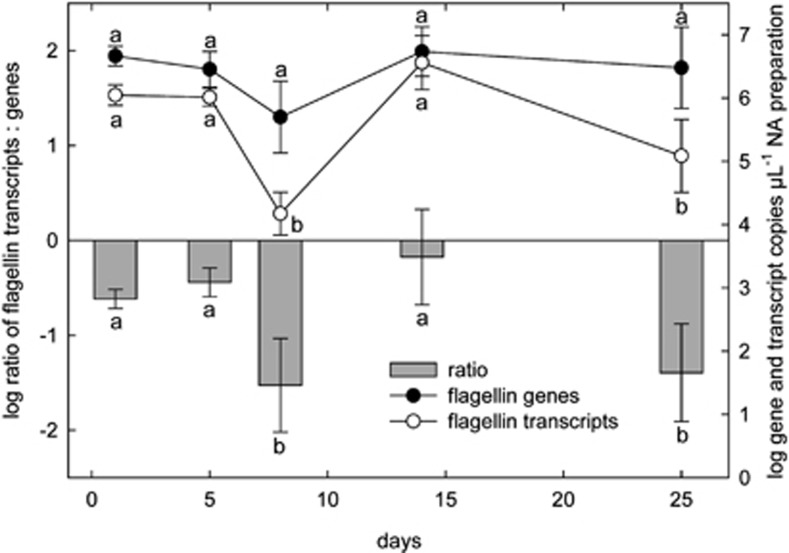
Clostridial flagellin transcript and gene copies during colitis and recovery. RNA and DNA were recovered from the same nucleic acids preparation, RNA was reverse transcribed into single-stranded cDNA. Gene and transcript copies were determined with qPCR. The ratio of flagellin to gene copies was calculated for individual mice and log-transformed. Means were compared by One-Way ANOVA, different letters indicate significance (*P*<0.05).

The SEED category ‘Motility and Chemotaxis' consisted to a large part (80%) of transcripts of clostridial ‘Flagellar Motility'. ‘Motility and Chemotaxis' dominated the separation of functional clusters along the first principal component of the PCA plot and was also responsible for the large spread of data points during recovery phase (day 25) (Figure 3). At days 5 and 8, there were 21 and 49% less transcripts assigned to ‘Motility and Chemotaxis' compared to day 1. (Supplementary Figure S5). Likewise, the relative abundance of ‘Motility and Chemotaxis' was reduced in the metatranscriptomes of *Firmicutes* (Supplementary Figure S7). To determine whether this reduction that mirrored the decrease of *Clostridiales* and/or *Lachnospiraceae* 16S rRNA was due to species reorganization within the phyla *Firmicutes* or was caused by a downregulation of flagellin gene expression, we additionally determined abundances of clostridial flagellin genes and transcripts by qPCR (Figure 5). Flagellin genes and transcripts were reduced at day 8 compared to day 1, and flagellin transcripts were also lower at day 25 than at the start of the experiment. Yet, whereas at days 1 and 5 the ratio of transcripts to genes remained fairly stable, it was significantly reduced at days 8 and 25, and there was a strong variation in replicates at day 14 (Figure 5) hinting that the observed reduction of transcripts during acute colitis and at day 25 was not only caused by the reduced abundance of *Lachnospiraceae* but was also caused by a downregulation of flagellin transcription.

To further validate the decreased expression of clostridial flagellin genes during acute colitis, we re-analyzed data from a previous experiment in a different genotype (STAT1^−/−^ mice on C57BL/6 background; Berry *et al.*, 2012), during which we had also observed a decrease of flagellins in metatranscriptomes. Also here, the ratio of flagellin transcripts to genes was reduced ∼fourfold (*P*<0.05) in mice suffering from acute colitis compared to a control group (control group (*n*=5): 0.09±0.18, colitis group (*n*=5): −0.34±0.31 [log ratio flagellin transcripts/genes]).

### Mucin degradation and utilization during acute colitis

**Figure 6:**
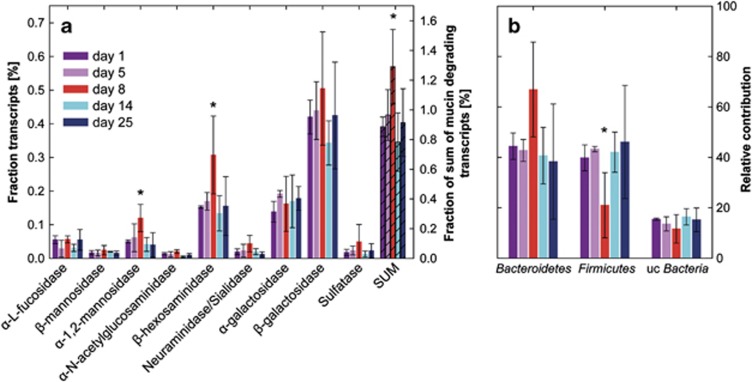
Mucin degradation-related transcripts during the development and recovery from colitis. (**a**) Fraction of transcripts related to mucin degradation in the metatranscriptomes. (**b**) Taxonomic assignment of transcripts related to mucin degradation. Means at days 1 and 8 were compared with unpaired *t*-test; *P*<0.05 were considered significantly different (*). uc, unclassified.

The SEED category ‘Aminosugars', which includes β-hexosaminidases capable of releasing N-acetylglucosamines and N-acetylgalactosamines from O-linked mucins, was significantly more abundant in inflamed mice than in healthy or recovering animals (Figure 4). That led us to investigate whether the transcription of glycoside hydrolases putatively involved in mucin degradation and sulfatases catalyzing the rate-limiting step of mucin degradation (Wright *et al.*, 2000) were affected by inflammation. Beta-mannosidase, neuraminidase/sialidase, β-galactosidase and sulfatase transcripts were more abundant at day 8 compared to day 1; α-mannosidase and β-hexosaminidase transcripts were significantly enriched (Figure 6). Taken together, transcripts of all genes presumably involved in mucin degradation were significantly higher on day 8 compared to day 1 (Figure 6). This increase corresponded to a significant increase of transcripts of mucin-degrading enzymes within the *Bacteroidetes* transcriptomes during inflammation (day 1: 1.36±0.05%, day 5: 1.39±0.26%, day 8: 2.03±0.28%, day 14: 1.31±0.44% and day 25: 1.33±0.11%).

Enhanced degradation of mucin could increase the availability of mucin components. Indeed, transcripts of fucose uptake and utilization were slightly more abundant while mannose and N-hexosaminidases uptake and utilization remained unaffected (Supplementary Table S6). Galactose uptake and metabolism was significantly reduced due to the decrease of the galactose/methyl galactoside ABC transport system MglABC (Supplementary Table S3). MglABC is a high-affinity ABC transporter acting as a scavenging system in case of low substrate concentration (Wilson, 1974), thus its downregulation suggested increased substrate availability.

### Microbiota-host networks in acute colitis and recovery

**Figure 7:**
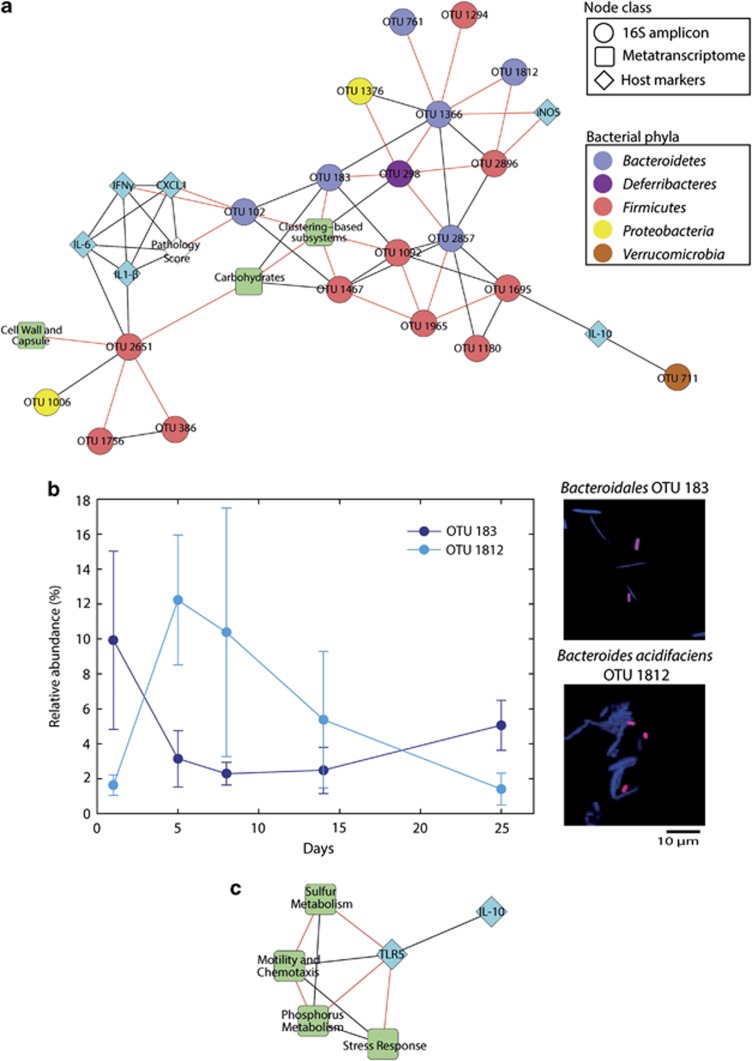
Correlation networks of 16S rRNA gene amplicon defined OTUs, functional categories (SEED subsystems level 1) and host parameters. (**a**) Sub-network of inflammation or pathology-associated host markers and their first and second neighbours in microbiota data sets. (**b**) Relative abundance of the inflammation-associated *Bacteroides acidifaciens* OTU 1812 and the health-associated *Bacteroidales* OTU 183 in amplicon sequencing data and representative fluorescence *in situ* hybridization images of the two phylotypes. (**c**) Sub-network of first neighbours of TLR5. Black and red edges indicate positive and negative correlations, respectively (*r*>0.6 or *r*<−0.6).

We used correlation networks to identify bacterial phylotypes associated with functional transcripts and host parameters. *IFNγ*, *IL-6*, *IL-1β* and *CXCL1* expression levels correlated with pathology scores, and members of this cluster correlated positively with *Turicibacteraceae* OTU 2651 and negatively with *Bacteroidales* OTU 102 (Figure 7a). Additionally, inducible nitric oxide synthase expression was negatively correlated with *Lachnospiraceae* OTU 2896 and *Bacteroidales* OTU 1366, and IL10 expression was positively correlated with *Ruminococcaceae* OTU 1695 and *Akkermansia* OTU 711. Examination of the first neighbours of phylotypes that correlated with host markers revealed extensive intra-phylum discordance in phylotype abundances in the *Bacteroidetes* and *Firmicutes*, for example, *Bacteroidales* OTU 183 decreased during inflammation and recovered by day 25, while *Bacteroides acidifaciens* OTU 1812 increased transiently during inflammation (Figure 7b), which highlights the inner-group dynamics occurring during dysbiosis. As clostridial flagellin transcription was heavily impacted during inflammation, we also quantified the expression of TLR5, the pattern recognition receptor of flagellins (data not shown). TLR5 correlated with ‘Motility and Chemotaxis' and the anti-inflammatory cytokine *IL-10* (Figure 7c).

## Discussion

A global perspective on microbial gene expression pattern before, during and after acute intestinal inflammation has been lacking. We thus performed a longitudinal study of the intestinal metatranscriptome and bacterial community dynamics, and selected host parameters in DSS-treated mice to address the following questions: How resistant and resilient is the gut microbiota to acute intestinal inflammation? Which microbial activities are affected during acute colitis and recovery? How are microbial energy and carbon metabolism impacted by the inflammation? What are the impacts of colitis on host–microbiota interactions?

### Host and microbiota during progression of DSS-triggered acute colitis and recovery

**Figure 8:**
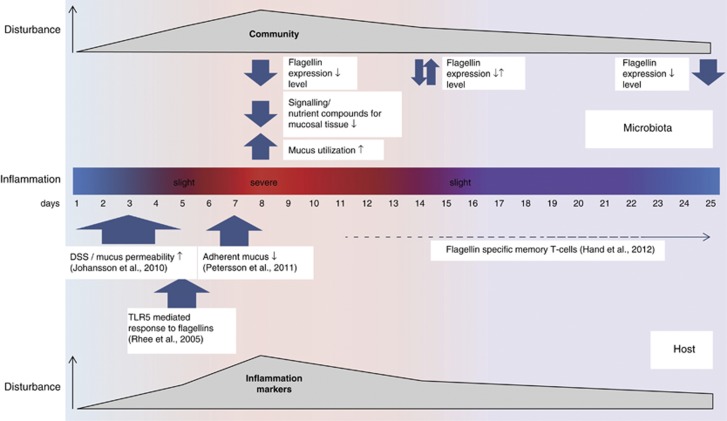
Timeline of the interplay of host and microbiota during colitis and recovery.

DSS destroys the mucus biopolymer network resulting in enhanced mucus permeability; however, mucus biosynthesis is not affected (Johansson *et al.*, 2010). The loss of barrier function allows the translocation of microbes, leading to increased stimulation of epithelial immune cells and the induction of inflammatory responses (Kitajima *et al.*, 1999). We started to observe weight loss, tissue inflammation and the downregulation of *IL-10* known to limit the expression of cytokines from dendritic cells and macrophages after activation by TLRs (Murray, 2005) at day 5 (Figure 8). Microbiota alterations occurred concurrently with the onset of inflammation. Members of the *Bacteroidetes* were identified as possible indicators of disease onset in this study, and were previously shown to be capable of inducing colitis in antibiotic-pretreated mice (Bloom *et al.*, 2011). However, abundance shifts of *Firmicutes* and *Bacteroidetes* and intra-phylum variations only had a minor impact on microbiota gene expression at day 5, highlighting a certain degree of functional redundancy of the microbial community in the face of slight perturbations. The significant upregulation of markers of inflammation, extensive weight loss and high inflammation scores marked the onset of full-blown colitis (day 8).

Confirming previous studies, acute colitis was characterised by a distinctive dysbiosis (Frank *et al.*, 2007; Nagalingam *et al.*, 2011; Berry *et al.*, 2012) manifested by an increase of low abundant families and concurrent intra- and inter-phyla abundance shifts of the *Bacteroidetes* and *Firmicutes*.

Acute colitis likewise had major impacts on gene expression indicated by temporal abundance changes of mRNA belonging to broad functional categories. With the recovery of the host from acute inflammation, severely disturbed microbial traits contributing to host–microbiota interactions, such as butyrate formation and mucin utilization recovered quickly to pre-inflammation levels (day 14). In contrast, community composition was characterised by a memory of disturbance comparable to that observed in studies using antibiotics as disturbing agents (De La Cochetière *et al.*, 2005, Dethlefsen and Relman, 2011). Upregulation of pro-inflammatory cytokines, inflamed tissue and a compositionally altered microbiota persisted up to day 25. In this state, the interdependent host–microbiota system might remain sensitive towards relapsing flares of inflammation.

Correlation networks were produced in order to compare changes in the microbiota with inflammation/immune state. This analysis revealed that pathology scores and expression of pro-inflammatory cytokines correlated and also were correlated (both positively and negatively) with several bacterial phylotypes (Figure 7a). With future validation, this approach of identifying gut bacterial phylotypes that correlate with host health markers may be a valuable tool for the development of novel non-invasive bacterial biomarkers of intestinal disease and may reduce the need for endoscopy.

### Impact of acute colitis on *Clostridiales*–host interactions

Increasing evidence points to an errant immune response directed at commensals during acute inflammation (Hand *et al.*, 2012); one frequently identified potential target molecule is the flagellin of *Clostridiales* (Lodes *et al.*, 2004; Duck *et al.*, 2007; Hand *et al.*, 2012). A decrease of *Clostridiales* during acute colitis and the concurrent reduction of clostridial flagellins as described in this study has been repeatedly observed in humans and in animal models (Frank *et al.*, 2007; Erridge *et al.*, 2010; Berry *et al.*, 2012), but the underlying causes have received little attention. Upon disruption of the mucus layer and epithelium by DSS treatment, the basolateral flagellin receptor TLR5 (Hayashi *et al.*, 2001) and intracellular flagellin sensor NLRC4 (Lightfield *et al.*, 2008) can be stimulated (Figure 8, Rhee *et al.*, 2005), eventually leading to specific antibody production (Vijay-Kumar *et al.*, 2010b). Indeed, we observed a correlation between relative abundance of flagellin transcripts and expression of TLR5 (Figure 7c). Vijay-Kumar *et al.*, 2010a previously showed the regulatory impact of TLR5 on the gut microbiota as its inactivation resulted in increased bacterial load and changes in *Firmicutes* and *Bacteroidetes* species composition. In colitic mice and human IBD patients, antibodies targeting flagellins of commensal strains were dominant antigens (Lodes *et al.*, 2004; Duck *et al.*, 2007). This directed immune response against flagellated members of the major intestinal phylum *Firmicutes* might be one reason for the observed abundance shift of *Clostridiales* and *Bacteroidetes* in acute colitis.

The downregulation of flagellin transcription (Figure 5) observed here and in a previous study hints that members of *Lachnospiraceae* have developed a strategy to avoid, at least to some extent, attacks by the host in a similar way as pathogens that tightly regulate flagellin transcription to maintain low metabolic costs and to escape recognition by the host immune system (Winter *et al.*, 2010). In *Bacteroides thetaiotaomicron*, the expression of a carbohydrate epitope was observed to negatively correlate with IgA levels, indicating that commensals are capable of modifying their surface structure in response to host activity (Peterson *et al.*, 2007). Disturbances in flagellin expression could still be observed during the recovery phase. This is in line with observations that clostridial flagellin-specific memory T-cells that were initiated during acute colitis caused by *T. gondii* infection or DSS, persisted for more than two months and could be rapidly activated in case of another challenge (Hand *et al.*, 2012).

### Impact of acute colitis on *Bacteroidetes*-host interactions

In healthy mice, the phyla *Firmicutes* and *Bacteroidetes* both contributed to transcripts associated with enzymes involved in degradation of mucin glycoproteins. During inflammation, the *Bacteroidetes* might be the numerically abundant group of mucin degraders, as judged from an increase in their relative abundance and the expression of the respective transcripts. Increased abundance of transcripts related to mucus degradation was contrasted by decreased abundance of ‘Polysaccharide Metabolism', possibly indicating a shift in substrate preference within the *Bacteroidetes*. This shift was likely due to shifting intra-phylum composition because Bray–Curtis dissimilarity, correlation network analysis and FISH revealed that this shift was not a common event in all *Bacteroidetes* phylotypes, but rather that a subgroup profited from the inflammation-induced changes in the intestinal milieu. One of the phylotypes enriched during acute colitis was *Bacteroides acidifaciens* (OTU 1812, Figure 7b), a species originally isolated from mouse cecum (Miyamoto and Itoh, 2000). *B. acidifaciens* was also previously identified as an indicator phylotype in acute colits (Berry *et al.*, 2012). *B. acidifaciens,* together with *Akkermansia,* was recently described as an important mucin-degrader *in vivo* (Berry *et al.*, 2013). In the present study, the increase of *B. acidifaciens* was correlated positively with several mucin glycoprotein-degrading enzyme transcripts (α-1,2-mannosidase, *r*: 0.683, β-hexosaminidase, *r*: 0.713, sialidase, *r*: 0.551, *P*<0.05), suggesting that this strain, as a mucin degrader, might have profited from the changed microenvironment during acute colitis.

In contrast to *B. acidifaciens,* the metatranscriptomic analysis did not provide indications for *Akkermansia* as mucus degrader during inflammation, although this organism possesses all genomic features to degrade complex mucus components (van Passel *et al.*, 2011). Possibly, the sequencing depth of the metatranscriptome was not sufficient for such an identification, since *Akkermansia* abundances were very low in the mice analyzed.

### Temporary alterations of metabolic interactions between microbiota and host during acute colitis

Abundance and intra-taxon shifts of *Clostridiales* and *Bacteroidales* during the acute phase of inflammation have far-reaching implications on metabolite-related host–microbiota interactions. The decrease in butyrate-producing clostridia indicated by the decrease of clostridial transcripts of pyruvate-formate lyase and key enzymes of butyrate fermentation pathways, potentially resulted in a reduction of butyrate production (Supplementary Figure S1, 4B, Supplementary Table S4). Concurrently, *Bacteroidetes* such as the mucolytic *B. acidifaciens* form acetate, succinate and potentially propionate as metabolic end products (Macy and Probst, 1979; Miyamoto and Itoh, 2000) shifting the balance of SCFA towards acetate and propionate. As acetate is absorbed and transported to the liver, a shifted SCFA balance can lead to a decrease in available butyrate as an energy source for colonic epithelial cells, but might also affect regulation of colonic cell proliferation and differentiation (Wong *et al.*, 2006; Plöger *et al.*, 2012). The high abundance of transcripts of pyruvate-formate lyase, an oxygen-sensitive enzyme that reversibly converts pyruvate to acetyl-CoA and formate in clostridia (Thauer *et al.*, 1972) emphasizes its role as a second major pyruvate metabolism pathway beside pyruvate:ferroxin oxidoreductase (Uyeda and Rabinowitz, 1971). Pyruvate-formate lyase was also prominent in the human intestinal metaproteome (Kolmeder *et al.*, 2012).

## Conclusion

In this study, gut microbiota activity and interactions with the host during acute inflammation and recovery were investigated. With the information gained, we proposed a time line of events that determined the interplay of host and microbiota (Figure 8). Inflammation temporarily had a large impact on the microbiota and the host; however, the host–microbiota homeostasis revealed a significant resilience, particularly evident in the transcriptional activity of the gut microbiota. While acute DSS colitis is certainly different from IBD, there are some similarities such as shift in microbiota composition with decrease of flagellated, butyrate producing *Lachnospiracae* and an increase of *Enterobacteriacae* (Frank *et al.*, 2007), as well as changes in mucus degrader activity (Png *et al.*, 2010). Therefore, the mechanistic outcome of this study might be relevant for research on human IBD.

## References

[bib1] Atarashi K Tanoue T Shima T Imaoka A Kuwahara T Momose Y 2011 Induction of colonic regulatory T cells by indigenous Clostridium species Sci 331 337 341 10.1126/science.1198469 PMC3969237 21205640

[bib2] Bergman EN 1990 Energy contributions of volatile fatty acids from the gastrointestinal tract in various species Physiol Rev 70 567 590 2181501 10.1152/physrev.1990.70.2.567

[bib3] Berry D Reinisch W 2013 Intestinal microbiota: a source of novel biomarkers in inflammatory bowel disease? Best Pract Res Clin Gastroenterol 27 47 58 10.1016/j.bpg.2013.03.005 23768552

[bib4] Berry D Schwab C Milinovich G Reichert J Ben Mahfoudh K Decker T 2012 Phylotype-level 16S rRNA analysis reveals new bacterial indicators of health state in acute murine colitis ISME J 6 2091 2106 22572638 10.1038/ismej.2012.39 PMC3475367

[bib5] Berry D Stecher B Schintlmeister A Reichert J Brugiroux S Wild B 2013 Host-compound foraging by intestinal microbiota revealed by single-cell stable isotope probing Proc Natl Acad Sci USA 110 4720 4725 23487774 10.1073/pnas.1219247110 PMC3607026

[bib6] Bloom SM Bijanki VN Nava GM Sun L Malvin NP Donermeyer DL 2011 Commensal Bacteroides species induce colitis in host-genotype-specific fashion in a mouse model of inflammatory bowel disease Cell Host Microbe 9 390 403 21575910 10.1016/j.chom.2011.04.009 PMC3241010

[bib7] Caporaso JG Kuczynski J Stombaugh J Bittinger K Bushman FD Costello EK 2010 QIIME allows analysis of high-throughput community sequencing data Nat Methods 7 335 336 20383131 10.1038/nmeth.f.303 PMC3156573

[bib8] De La Cochetière MF Durand T Lepage P Bourreille A Galmiche JP Doré J 2005 Resilience of the dominant human fecal microbiota upon short-course antibiotic challenge J Clin Microbiol 43 5588 5592 16272491 10.1128/JCM.43.11.5588-5592.2005 PMC1287787

[bib9] Dethlefsen L Relman DA 2011 Incomplete recovery and individualized responses of the human distal gut microbiota to repeated antibiotic perturbation Proc Natl Acad Sci USA 108 4554 4561 20847294 10.1073/pnas.1000087107 PMC3063582

[bib10] Duck LW Walter MR Novak J Kelly D Tomasi M Cong Y 2007 Isolation of flagellated bacteria implicated in Crohn's Disease Inflamm Bowel Dis 13 1191 1201 17712838 10.1002/ibd.20237

[bib11] Erickson AR Cantarel BL Lamendella R Darzi Y Mongodin EF Pan C 2012 Integrated metagenomics/metaproteomics reveals human host-microbiota signatures of Crohn's Disease PLoS One 7 e49138 23209564 10.1371/journal.pone.0049138 PMC3509130

[bib12] Erridge C Duncan SH Bereswill S Heimesaat MM 2010 The induction of colitis and ileitis in mice is associated with marked increases in intestinal concentrations of stimulants of TLRs 2, 4, and 5 PLoS One 5 e9125 20161736 10.1371/journal.pone.0009125 PMC2817728

[bib13] Frank DN St Amand AL Feldman RA Boedeker EC Harpaz N Pace NR 2007 Molecular-phylogenetic characterization of microbial community imbalances in human inflammatory bowel disease Proc Natl Acad Sci USA 104 13780 13785 17699621 10.1073/pnas.0706625104 PMC1959459

[bib14] Griffiths RI Whiteley AS O'Donnell AG Bailey MJ 2000 Rapid method for coextraction of DNA and RNA from natural environments for analysis of ribosomal DNA- and rRNA-based microbial community composition Appl Environ Microbiol 66 5488 5491 11097934 10.1128/aem.66.12.5488-5491.2000 PMC92488

[bib15] Hammer Ø Harper DAT Ryan PD 2001 PAST: paleontological statistics software package for education and data analysis Palaeontologia Electronica 4 9

[bib16] Hand TW Dos Santos LM Bouladoux N Molloy MJ Pagan AJ Pepper M 2012 Acute gastrointestinal infection induces long-lived microbiota-specific T cell responses Sci 337 1553 1556 10.1126/science.1220961 PMC3784339 22923434

[bib17] Hayashi F Smith KD Ozinsky A Hawn TR Yi EC Goodlett DR 2001 The innate immune response to bacterial flagellin is mediated by Toll-like receptor 5 Nat 410 1099 1103 10.1038/35074106 11323673

[bib18] Helbling DE Ackermann M Fenner K Kohler H-PE Johnson DR 2012 The activity level of a microbial community function can be predicted from its metatranscriptome ISMEJ 6 902 904 10.1038/ismej.2011.158 PMC3309364 22094344

[bib19] Honda K Littman DR 2012 The microbiome in infectious disease and inflammation Ann Rev Immunol 30 759 795 22224764 10.1146/annurev-immunol-020711-074937 PMC4426968

[bib20] Horino J Fujimoto M Terabe F Serada S Takahashi T Soma Y 2008 Suppressor of cytokine signaling-1 ameliorates dextran sulfate sodium-induced colitis in mice Int Immunol 20 753 762 18381351 10.1093/intimm/dxn033

[bib21] Johansson MEV Gustafsson JK Sjöberg K Petersson J Holm L Sjövall H 2010 Bacteria penetrate the inner mucus layer before inflammation in the dextran sulfate colitis model PLoS One 5 e12238 20805871 10.1371/journal.pone.0012238 PMC2923597

[bib22] Kitajima S Takuma S Morimoto M 1999 Changes in colonic mucosal permeability in mouse colitis induced with dextran sulfate sodium Exp Anim 48 137 143 10480018 10.1538/expanim.48.137

[bib23] Kolmeder CA de Been M Nikkilä J Ritamo I Mättö J Valmu L 2012 Comparative metaproteomics and diversity analysis of human intestinal microbiota testifies for its temporal stability and expression of core functions PLoS One 7 e29913 22279554 10.1371/journal.pone.0029913 PMC3261163

[bib24] Kumar H Kawai T Akira S 2009 Toll-like receptors and innate immunity Biochem Biophys Res Comm 388 621 625 19686699 10.1016/j.bbrc.2009.08.062

[bib25] Lane DJ 1991 16S/23S rRNA sequencingIn Stackebrandt E, Goodfellow M (eds.)Nucleic Acid Techniques in Bacterial Systematics John Wiley & Sons: Chichester 115 175

[bib26] Lanzén A Jørgensen SL Huson DH Gorfer M Grindhaug SH Jonassen I 2012 CREST – Classification resources for environmental sequence tags PLoS One 7 e49334 23145153 10.1371/journal.pone.0049334 PMC3493522

[bib27] Lightfield KL Persson J Brubaker SW Witte CE von Moltke J Dunipace EA 2008 Critical function for Naip5 in inflammasome activation by a conserved carboxy-terminal domain of flagellin Nat Immunol 9 1171 1178 18724372 10.1038/ni.1646 PMC2614210

[bib28] Lodes MJ Cong Y Elson CO Mohamath R Landers CJ Targan SR 2004 Bacterial flagellin is dominant antigen in Crohn disease J Clin Invest 113 1296 1306 15124021 10.1172/JCI20295 PMC398429

[bib29] Louis P Flint HJ 2009 Diversity, metabolism and microbial ecology of butyrate-producing bacteria from the human large intestine FEMS Microbiol Lett 294 1 8 19222573 10.1111/j.1574-6968.2009.01514.x

[bib30] Loy A Horn M Wagner M 2003 probeBase: an online resource for rRNA-targeted oligonucleotide probes Nucl Acids Res 31 514 516 12520066 10.1093/nar/gkg016 PMC165463

[bib31] Macy JM Ljungdahl LG Gottschalk G 1978 Pathway of succinate and propionate formation in Bacteroides fragilis J Bacteriol 134 84 91 148460 10.1128/jb.134.1.84-91.1978 PMC222221

[bib32] Macy JM Probst I 1979 The biology of gastrointestinal Bacteroides Ann Rev Microbiol 33 561 594 386933 10.1146/annurev.mi.33.100179.003021

[bib33] Magoč T Salzberg SL 2011 FLASH: fast length adjustment of short reads to improve genome assemblies Bioinformatics 27 2957 2963 21903629 10.1093/bioinformatics/btr507 PMC3198573

[bib34] Mitra S Stärk M Huson DH 2011 Analysis of 16S rRNA environmental sequences using MEGAN BMC Genomics 12 S17 22369513 10.1186/1471-2164-12-S3-S17 PMC3333176

[bib35] Morgan XC Tickle TL Sokol H Gevers D Devaney KL Ward DV 2012 Dysfunction of the intestinal microbiome in inflammatory bowel disease and treatment Genome Biol 13 R79 23013615 10.1186/gb-2012-13-9-r79 PMC3506950

[bib36] Murray PJ 2005 The primary mechanism of the IL-10-regulated anti-inflammatory response is to selectively inhibit transcription Proc Natl Acad Sci USA 102 8686 8691 15937121 10.1073/pnas.0500419102 PMC1150817

[bib37] Miyamoto Y Itoh K 2000 Bacteroides acidifaciens sp. nov., isolated from the caecum of mice Int J Sys Evol Microbiol 50 145 148 10.1099/00207713-50-1-145 10826798

[bib38] Nagalingam NA Phil M Kao JY Young VB 2011 Microbial ecology of the murine gut associated with the development of DSS-colitis Inflamm Bowel Dis 17 917 926 21391286 10.1002/ibd.21462 PMC3058753

[bib39] Nicholson JK Holmes E Kinross J Burcelin R Gibson G Jia W 2012 Host-gut microbiota metabolic interactions Sci 336 1262 1267 10.1126/science.1223813 22674330

[bib40] Okayasu I Hatakeyama S Yamada M Ohkusa T Inagaki Y Nakaya R 1990 A novel method in the induction of reliable experimental acute and chronic ulcerative colitis in mice Gastroenterol 98 694 702 10.1016/0016-5085(90)90290-h 1688816

[bib41] Peterson DA McNulty NP Guruge JL Gordon JI 2007 IgA response to symbiotic bacteria as a mediator of gut homeostasis Cell Host Microbe 2 328 339 18005754 10.1016/j.chom.2007.09.013

[bib42] Pfaffl MW Horgan GW Dempfle L 2002 Relative expression software tool (REST©) for group-wise comparison and statistical analysis of relative expression esults in real-time PCR Nucl Acids Res 30 e36 11972351 10.1093/nar/30.9.e36 PMC113859

[bib43] Plöger S Stumpff F Penner GB Schulzke J-D Gäbel G Martens H 2012 Microbial butyrate and its role for barrier function in the gastrointestinal tract Ann NY Acad Sci 1258 52 59 22731715 10.1111/j.1749-6632.2012.06553.x

[bib44] Png CW Lindén SK Glishenan KS Zoetendal EG McSweeney CS Sly LI 2010 Mucolytic bacteria with increased prevalence in IBD mucosa augment *in vitro* utilization of mucin by other bacteria Am J Gastroenterol 105 2420 2428 20648002 10.1038/ajg.2010.281

[bib45] Pryde SE Duncan SH Hold GL Stewart CS Flint HJ 2006 The microbiology of butyrate formation in the human colon FEMS Microbiol Lett 217 133 139 12480096 10.1111/j.1574-6968.2002.tb11467.x

[bib46] Quast C Pruesse E Yilmaz P Gerken J Schweer T Yarza P 2013 The SILVA ribosomal RNA gene database project: improved data processing and web-based tools Nucl Acids Res 41 D590 D596 23193283 10.1093/nar/gks1219 PMC3531112

[bib47] Rhee SH Im E Riegler M Kokkotou E O'Brien M Pothoulakis C 2005 Pathophysiological role of Toll-like receptor 5 engagement by bacterial flagellin in colonic inflammation PNAS 102 13610 13615 16157881 10.1073/pnas.0502174102 PMC1224619

[bib48] Saleh M Trinchieri G 2011 Innate immune mechanism of colitis and colitis associated colorectal cancer Nat Rev Immunol 11 9 20 21151034 10.1038/nri2891

[bib49] Schloss PD Gevers D Westcott SL 2011 Reducing the effects of PCR amplification and sequencing artifacts on 16S rRNA-based studies PLoS One 6 e27310 22194782 10.1371/journal.pone.0027310 PMC3237409

[bib50] Schramm A Fuchs BM Nielsen JL Stahl DA 2002 Fluorescence *in situ* hybridization of 16S rRNA gene clones (Clone-FISH) for probe validation and screening of clone libraries Environ Microbiol 4 713 720 12460279 10.1046/j.1462-2920.2002.00364.x

[bib51] Shannon P Markiel A Ozier O Baliga NS Wang JT Ramage D 2003 A software environment for integrated models of biomolecular interaction networks Genome Res 13 2498 2504 14597658 10.1101/gr.1239303 PMC403769

[bib52] Stecher B Berry D Loy A 2013 Colonization resistance and microbial ecophysiology: using gnotobiotic mouse models and single-cell technology to explore the intestinal jungle FEMS Microbiol Rev 37 793 829 23662775 10.1111/1574-6976.12024

[bib53] Stockinger S Reutterer B Schaljo B Schellack C Brunner S Materna T 2004 IFN regulatory factor 3-dependent induction of type I IFNs by intracellular bacteria is mediated by a TLR- and Nod2-independent mechanism J Immunol 173 7416 7425 15585867 10.4049/jimmunol.173.12.7416

[bib54] Thauer RK Kirchniawy H Jungermann KA 1972 Properties and function of the pyruvate-formate-lyase reaction in Clostridiae Eur J Biochem 27 282 290 4340563 10.1111/j.1432-1033.1972.tb01837.x

[bib55] Urich T Lanzén A Qi J Huson DH Schleper C Schuster SC 2008 Simultaneous assessment of soil microbial community structure and function through analysis of the meta-transcriptome PLoS One 3 e2527 18575584 10.1371/journal.pone.0002527 PMC2424134

[bib56] Uyeda K Rabinowitz JC 1971 Pyruvate-ferredoxin oxidoreductase. III Purification and properties of the enzyme J Biol Chem 246 3111 3119 5574389

[bib57] van Passel MWJ Kant R Zoetendal EG Plugge CM Derrien M Malfatti SA 2011 The genome of Akkermansia muciniphila, a dedicated intestinal mucin degrader, and its use in exploring intestinal metagenomes PLoS One 6 e16876 21390229 10.1371/journal.pone.0016876 PMC3048395

[bib58] Vickery BP Scurlock AM Jones SM Wesley-Burks A 2011 Mechanism of immune tolerance relevant to food allergy J Allergy Clin Immunol 127 576 584 21277624 10.1016/j.jaci.2010.12.1116 PMC3233381

[bib59] Vijay-Kumar M Aitken JD Carvalho FS Cullender TC Mwangi S Srinivasan S 2010 Metabolic syndrome and altered gut microbiota in mice lacking Toll-Like Receptor 5 Sci 328 228 231 10.1126/science.1179721 PMC4714868 20203013

[bib60] Vijay-Kumar M Carvalho FA Aitken JD Fifadara NH Gerwitz AT 2010 TLR5 or NLRC4 is necessary and sufficient for promotion of humoral immunity by flagellin Eu J Immunol 40 3528 3534 10.1002/eji.201040421 PMC3081661 21072873

[bib61] Wang JMW de Souza R Kendall CWC Emam A Jenkins DJA 2006 Colonic health: fermentation and short chain fatty acids J Clin Gastroenterol 40 235 243 16633129 10.1097/00004836-200603000-00015

[bib62] Wilson DB 1974 The regulation and properties of the galactose transport system in Escherichia coli K12 J Biol Chem 249 553 558 4588568

[bib63] Winter SE Winter MG Godinez I Yang H-J Rüssmann H Andrews-Polymenis HL 2010 A rapid change in virulence gene expression during the transition from the intestinal lumen into tissue promotes systemic dissemination of Salmonella PLoS Pathog 6 e1001060 20808848 10.1371/journal.ppat.1001060 PMC2924370

[bib64] Wright DP Rosendale DI Robertson AM 2000 Prevotella enzymes involved in mucin oligosaccharide degradation and evidence for a small operon of genes expressed during growth on mucin FEMS Microbiol Lett 190 73 79 10981693 10.1111/j.1574-6968.2000.tb09265.x

