# Supplementary material for: Longitudinal study of murine microbiota activity and interactions with the host during acute inflammation and recovery
Source: ISME J. 2014 Jan 9;8(5):1101–14. doi: 10.1038/ismej.2013.223 (PMC3996699; doi:10.1038/ismej.2013.223)
Supplement: Supplementary Information [file ismej2013223x1.doc]

**Longitudinal study of murine microbiota activity and interactions with the host during acute inflammation and recovery**

Clarissa Schwab^1^, David Berry^2^, Isabella Rauch^3^, Ina Rennisch^1^, Julia Ramesmayer^2^, Eva Hainzl^4^, Susanne Heider^5^, Thomas Decker^3^, Lukas Kenner^5^, Mathias Müller^4^, Birgit Strobl^4^, Michael Wagner^2^, Christa Schleper^1^, Alexander Loy^2^*, Tim Urich^1^*

^1^Division of Archaea Biology and Ecogenomics, Department of Ecogenomics and Systems Biology, Faculty of Life Sciences, University of Vienna, Althanstrasse 14, A-1090 Wien, Austria

^2^Division of Microbial Ecology, Department of Microbiology and Ecosystem Science, Faculty of Life Sciences, University of Vienna, Althanstrasse 14, A-1090 Wien, Austria

^3^Department of Microbiology, Immunobiology and Genetics, Max F. Perutz Laboratories, University of Vienna, Dr. Bohr-Gasse 9, A-1030 Wien, Austria

^4^Institute of Animal Breeding and Genetics, University of Veterinary Medicine Vienna, Veterinärplatz 1, A-1210 Wien, Austria

^5^Ludwig Boltzmann Institute for Cancer Research, Medical University of Vienna, Währingerstrasse13a, A-1090 Wien, Austria

*Corresponding authors: Tim Urich (Tel: +43 1 4277 76513, tim.urich@univie.ac.at) and Alexander Loy (Tel: +43 1 4277 76605, loy@microbial-ecology.net)

**Titles and legends to supplementary figures**

Figure S1. Shifts in bacterial communities during colitis and recovery. Samples were obtained from healthy mice (day 1), after DSS treatment (day 5), during acute colitis (day 8) and during recovery (days 14 and 25). 16S rRNA transcripts derived from metatranscriptome analysis were taxomomically assigned using MEGAN. (A) Relative abundance of 16S rRNA transcripts assigned to the predominating orders *Clostriales* and *Bacteroidales*. (B) ratio of *Clostridiales* and *Bacteroidales* 16S rRNA transcripts during the experiment*.* (C) Time course of relative 16S rRNA transcript abundance of dominant bacterial families and unclassified *Bacteroidales*. (D) Time course of 16S rRNA transcripts of minor bacterial families varying in abundance during colitis and recovery. uc, unclassified.

**Figure S2.** Beta-diversity of microbial communities during colitis and recovery. Shown are unweighted (A,C) and weighted Unifrac (B,D).

**Figure S3. Alpha diversity metrics for all samples**. Metrics are computed with 1000 bootstraps at 3,500 reads, and the arithmetic averages and 95% confidence intervals from biological replicates for each sampling day are shown. Metrics listed describe the phylotype richness ('Observed OTUs' and 'Chao1 Richness'), phylotype diversity and evenness ('Simpson Index', 'Shannon Index', and 'Equitability'), and sampling coverage ('Coverage'). n=4-5.

**Figure S4. Bray-Curtis dissimilarity**. A value of 1 indicates that the microbiomes of mice at different health states do not share any species (OTUs), whereas a of 0 shows that the species composition is the same. Compositional dissimimilarity of the bacterial population in samples obtained during the time-course of the study was investigated using Bray-Curtis dissimilarity based on relative abundance (upper panel) or presence and absence (lower panel) of OTUs.

**Figure S5.** **Relative abundance of SEED categories within mice metatranscriptomes during the course of the experiment.**  (A) Major SEED categories (B) Minor SEED categories. During acute colitis, ‘Motility and Chemotaxis’ (subcategory ‘Flagellar motility in Prokaryota’) was significantly reduced compared to day 1, while ‘RNA Metabolism’ (subcategories ‘Transcription’ and ‘RNA processing and modification’), ‘Clustering-based subsystems’ (subcategory ‘Bacterial Cell Division’), ‘DNA Metabolism’ (subcategories ‘DNA metabolism’ and ‘DNA replication’), ‘Cofactors, Vitamins, Prosthetic Groups, Pigments’ (subcategories ‘NAD and NADP’ and ‘Pyridoxine’), ‘Fatty Acids’ (subcategory ‘Fatty Acid Biosynthesis FASII’) significantly increased compared to day 1. Asterisk (*) indicate significant difference (p<0.05) between day 1 and 8.

**Figure S6.** **Gene expression of *Bacteroidetes* in the mice guts during the experiment.** Fraction of *Bacteroidetes* transcripts assigned to major (A) and minor (B) SEED categories in the *Bacteroidetes* metatranscriptome. Fraction of *Bacteroidetes* transcripts assigned to major (C) and minor (D) categories in the metatranscriptome. Means at days 1 and 8 were compared with unpaired t-test, p<0.05 was considered significantly different (*).

**Figure S7.** **Gene expression of *Firmicutes* in the mice guts during the experiment.** Fraction of *Firmicutes* transcripts assigned to major (A) and minor (B) SEED categories in the *Firmicutes* metatranscriptome. Fraction of *Firmicutes* transcripts assigned to major (C) and minor (D) categories in the metatranscriptome. Means at days 1 and 8 were compared with unpaired t-test, p<0.05 was considered significantly different (*).

**Figure S8. ‘Carbohydrate Metabolism’ of *Bacteroidetes* and *Firmicutes*** in the mice guts during the experiment***.* (**A,C) Fraction of *Bacteroidetes* transcripts assigned to the category ‘Carbohydrate Metabolism’ in the *Bacteroidetes* metatranscriptome (A) or the entire metatranscriptome (C). (B,D) Fraction of *Firmicutes* assigned to ‘Carbohydrate Metabolism’ in the *Firmicutes* metatranscriptome (B) or the entire metatranscriptome (D). Means at days 1 and 8 were compared with unpaired t-test, p<0.05 was considered significantly different (*).

**Figure S9. Quantification of butyryl-CoA transferase genes and transcripts.** (A) Kinetic of ButCoA transferase genes and transcripts during the course of the experiment. Log transcript copies were not determined at day 5 and 25. (B) Log ButCoA transferase gene copies during a challenge of C57BL/6 mice with 3% DSS for five days. Mice lost weight a similar to mice treated with 2% DSS. (C) Graph showing both the decrease of ButCoA transcripts after 2 and 3% DSS treatment at day 8.

Figure S1.

**Figure S2.**


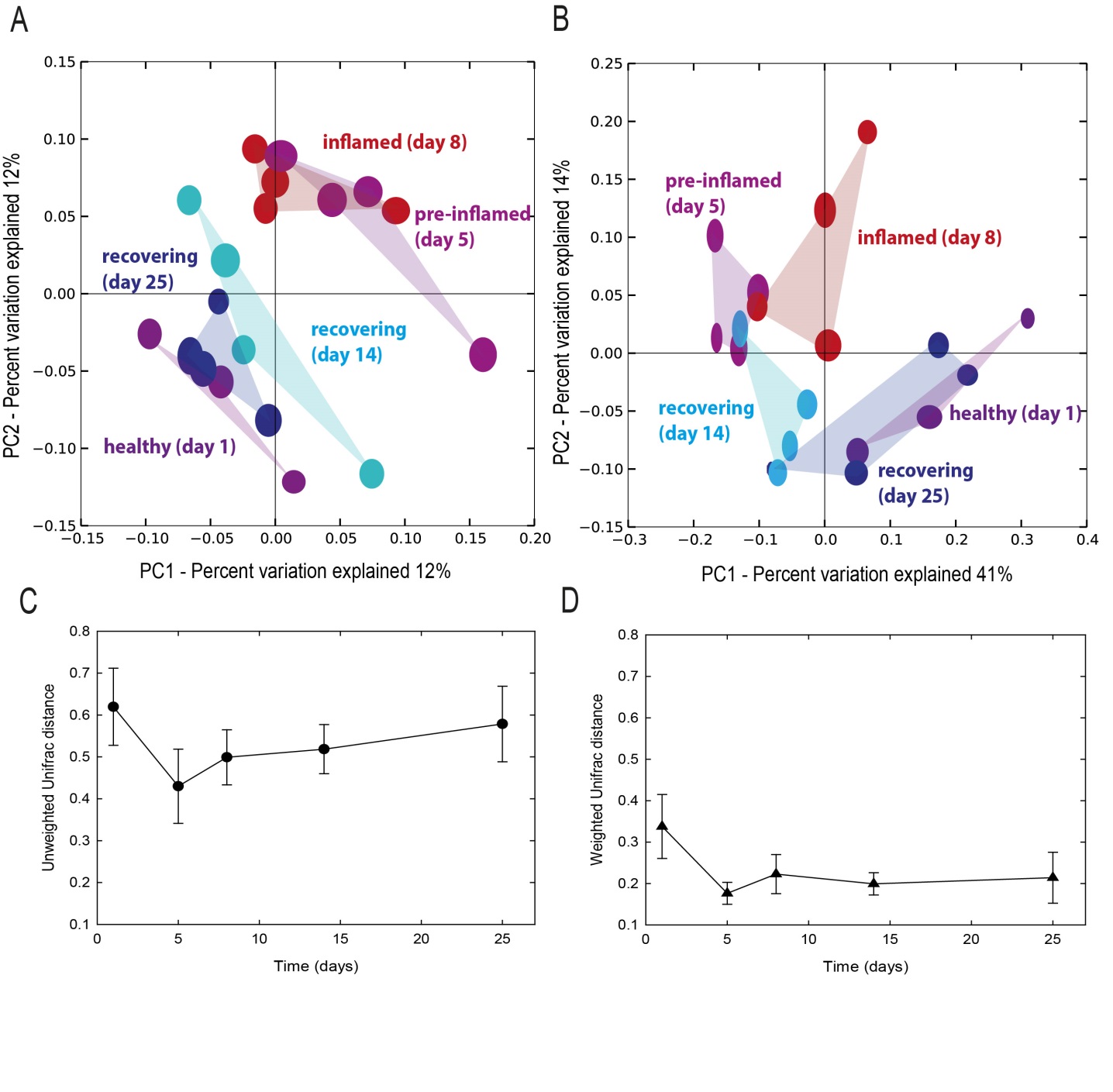


**Figure S3.**

**Figure S4.**

**Figure S5.**

**Figure S6.**

**Figure S7.**

**Figure S8.**

**Figure S9**.

**Table S1.** **Metatranscriptome sequencing.** Double stranded cDNA libraries were paired-end sequenced using an Illumina HiSeq (Campus Science Support Facilities GmbH, Vienna). Read pairs were overlapped using FLASH, which yielded reads of approx. 170 bp (Magoč and Salzberg, 2011). Metatranscriptomic sequencing data were analyzed following an established double RNA analysis pipeline (Urich et al., 2008).

| **Sampling Day** | **Mouse/**  **Sample ID** | **Overlapped fasta reads** | **rRNA reads** | **Putative mRNA-reads (%)** | **Putative mRNA functionally annotated in SEED (%)** |
| --- | --- | --- | --- | --- | --- |
| 1 | 1 | 5.126.605 | 4.892.326 | 234.279 (4.6) | 82.315 (35.1) |
|  | 2 | 8.377.912 | 7.839.163 | 538.749 (6.4) | 177.048 (32.9) |
|  | 3 | 4.173.904 | 3.979.306 | 194.598 (4.7) | 57.939 (29.8) |
| 5 | 9 | 13.656.219 | 12.489.289 | 1.166.930 (8.5) | 368.950 (31.6) |
|  | 10 | 5.212.686 | 4.841.497 | 371.189 (7.1) | 126.970 (34.2) |
|  | 11 | 9.982.257 | 9.622.069 | 360.188 (3.6) | 108.673 (30.2) |
| 8 | 17 | 3.049.523 | 2.974.218 | 75.305 (2.5) | 24.190 (32.1) |
|  | 18 | 5.819.226 | 5.687.909 | 131.317 (2.3) | 29.345 (22.3) |
|  | 19 | 9.257.790 | 9.054.252 | 203.538 (2.2) | 45.659 (22.4) |
|  | 20 | 10.032.175 | 9.736.821 | 295.354 (2.9) | 73.824 (25.0) |
| 14 | 25 | 7.205.739 | 6.632.670 | 573.069 (8.0) | 218.651 (38.2) |
|  | 26 | 8.719.790 | 8.028.255 | 691.535 (7.9) | 245.380 (35.5) |
|  | 27 | 4.670.669 | 4.442.922 | 227.747 (4.9) | 72.167 (31.7) |
| 25 | 33 | 8.739.836 | 8.425.407 | 314.429 (3.6) | 107.515 (34.2) |
|  | 34 | 15.647.416 | 15.339.081 | 308.335 (2.0) | 42.211 (13.7) |
|  | 35 | 4.948.311 | 4.661.045 | 287.266 (5.8) | 102.628 (35.5) |
|  | 36 | 6.571.890 | 6.255.937 | 315.953 (4.8) | 77.823 (24.6) |

**Table S2**. **qPCR primers used.** Primer specificity was confirmed by cloning and sequencing of PCR amplicons.

| Target gene | **Name** | **Sequence (5’-3’)** | **Annealing temperature** | **Reference** |
| --- | --- | --- | --- | --- |
| Butyryl-CoA CoA transferase | For | GCIGAICATTTCACITGGAAYWSITGGCAYATG | 53 | Louis and Flint, 2007 |
|  | Rev | CCTGCCTTTGCAATRTCIACRAANGC |  |  |
| Clostridial flagellins^a^ | For | CAGAACMGRYTRGAGCAYACVAT | 50 | This study |
|  | Rev | TTACTGTAAGAGCTGAAGTACACCCTG |  | Duck et al., 2007 |

**^a^** For amplification of clostridial flagellin gene fragments, a forward primer was constructed based on a conserved amino acid stretch ‘QNRLEHT‘(AA395-401 of Fla2 of ***Lachnospiraceae bacterium* A4 (A7IZG5_**9FIRM), see also Duck et al., 2007) present in flagellins Fla2 and Fla3 of *Lachnospiraceae* A4, in flagelins Fla1 and Fla2 of *Lachnospiraceae* A14-2, and in flagellins of *Eubacterium rectale*. BLASTP search confirmed that ‘QNRLEHT’ was also conserved in flagellins of other *Clostridium, Roseburia, Butyrivibrio* and *Eubacterium* spp. This primer was combined with the reverse general clostridial flagellin primer designed by Duck et al. (2007) yielding an amplicon of approx. 180 bp.

**Table S3**. **qPCR primers used for mice expression studies.**

| Target gene | Name | Sequence (5’-3’) | Annealing temperature | Reference |
| --- | --- | --- | --- | --- |
| Glyceraldehyde 3-phosphate dehydrogenase (*GAPDH*) | For | CATGGCCTTCCGTGTTCCTA | 60 | Kernbauer et al., 2012 |
|  | Rev | GCGGCACGTCAGATCCA |  |  |
| Chemokine (C-X-C motif) ligand 7 (*CXCL7*) | For | CTGGGATTCACCTCAAGAACATC | 60 | Jamieson et al., 2013 |
|  | Rev | CAGGGTCAAGGCAAGCCTC |  |  |
| Interleukin 6 (*IL-6*) | For | TAGTCCTTCCTACCCCAATTTCC | 60 | Kernbauer et al., 2012 |
|  | Rev | TTGGTCCTTAGCCACTCCTTC |  |  |
| Interleukin 1β (*IL1- β*) | For | GCAACTGTTCCTGAACTCAACT | 60 | Ydens et al., 2012 |
|  | Rev | ATCTTTTGGGGTCCGTCAACT |  |  |
| Inducible nitric oxide synthases (*iNOS*) | For | GCTTGCCCCAACAGGAGAAG | 60 | Jamieson et al., 2013 |
|  | Rev | GCT GCC CGG AAG GTT TGT AC |  |  |
| Interferon γ (*IFN-γ*) | For | AAGTGGCATAGATGTGGAAG | 60 | Putz et al., 2012 |
|  | Rev | GAATGCATCCTTTTTCGCCT |  |  |
| Toll-like receptor 5 (*TLR5*) | For | GCAGGATCATGGCATGTCAAC | 60 | This study |
|  | Rev | ATCTGGGTGAGGTTACAGCCT |  | Shang et al., 2011 |
|  |  |  |  |  |

**Table S4.** Bodyweight (relative to day 1), inflammation severity and extent, crypt damage and area involved of individual mice. ND, not determined; muc, mucosa; sm, submucosa; mp, muscularis propria; subser, subserosa; in brackets, added scoring.

| Day | Mouse | % Bodyweight | Inflammation severity | Inflammation extents | Crypt damage | % areainvolved |
| --- | --- | --- | --- | --- | --- | --- |
| 5 | 9 | ND | Mild (2) | muc+sm (2) | Mild (2) | 3 |
|  | 10 | ND | Very mild (1) | Muc (1) | Very mild (1) | <1 |
|  | 11 | ND | Very mild (1) | Muc (1) | Very mild (1) | <1 |
|  | 12 | ND | Very mild (1) | Muc (1) | Very mild (1) | <1 |
| 8 | 17 | 89.4 | Focal severe (3) | muc+sm+mp+sub ser (4) | Focal severe (3) | 13 |
|  | 18 | 89.3 | Focal severe (3) | muc+sm (2) | Focal severe (3) | 14 |
|  | 19 | 98.1 | Very mild (1) | Muc (1) | Very mild (1) | 2 |
|  | 20 | 87.9 | Very mild (1) | Muc (1) | Very mild (1) | 7 |
| 14 | 25 | 103 | Very mild (1) | Muc (1) | Very mild (1) | 3 |
|  | 26 | 89 | Very mild (1) | Muc (1) | Very mild (1) | 2 |
|  | 27 | 105 | Very mild (1) | Muc (1) | Very mild (1) | 3 |
|  | 28 | 103 | Focal severe (3) | muc+sm (2) | Focal severe (3) | 6 |
| 25 | 33 | 121 | Very mild (1) | Muc (1) | Very mild (1) | <1 |
|  | 34 | 116 | Very mild (1) | Muc (1) | Very mild (1) | <1 |
|  | 35 | 112 | Mild (2) | muc+sm (2) | Mild (2) | 1 |
|  | 36 | 114 | Focal severe (3) | muc+sm (2) | Mild (2) | 5 |

**Table S5** Abundance of ‘Acetyl-CoA-fermentation-to-butyrate’ subcategories [in o/oo] in the metatrancriptome.

| **Functional category** | **Relative abundance [o/_oo_]** | |  |
| --- | --- | --- | --- |
|  | **Day 1** | **Day 8** | **Significant** |
| Acetyl-CoA Acetyltransferase | 2.47±0.80 | 0.97±0.55 | YES(p<0.05) |
| Acetyl-CoA reductase | 0.06±0.03 | 0.02±0.02 |  |
| 3-hydroxybutyryl-CoA dehydratase | 1.43±0.38 | 0.68±0.19 | YES(p<0.05) |
| 3-hydroxybutyryl-CoA dehydrogenase | 0.64±0.18 | 0.34±0.20 |  |
| 3-hydroxyacyl-CoA dehydrogenase | 0.01±0.02 | 0.01±0.02 |  |
| Enoyl-CoA hydratase | 0.07±0.03 | 0.07±0.06 |  |
| Butyryl-CoA dehydrogenase | 3.42±1.04 | 1.49±0.41 | YES(p<0.05) |
| Electron transfer flavoprotein α-subunit | 3.42±1.05 | 1.43±0.47 | YES(p<0.05) |
| Electron transfer flavoprotein β-subunit | 2.64±0.75 | 1.43±0.48 | YES(p<0.05) |
| Phosphate butyryltransferase | 0.58±0.08 | 0.42±0.21 |  |
| Butyrate kinase | 0.64±0.19 | 0.50±0.21 |  |

**Table S6.** Relative abundance of mucin component uptake and utilization systems putatively involved in mucin degradation (see also Figure 6 in main manuscript) [in o/oo].

| **Functional category** | **Relative abundance [o/_oo_]** | |  |
| --- | --- | --- | --- |
|  | **Day 1** | **Day 8** | **Significant** |
| **Mannose uptake and utilization** | **2.08±0.09** | **2.37±0.42** |  |
| PTS system, mannose-specific II (ABCD components) | 0.17±0.08 | 0.11±0.10 |  |
| Mannose-6-phosphate isomerase | 0.37±0.04 | 0.42±0.17 |  |
| Phosphomannomutase | 0.96±0.16 | 0.67±0.28 |  |
| Mannose-1-phosphate guanylyltransferase (GDP) | 0.57±0.11 | 1.09±0.16 | YES (p<0.05) |
| **L-fucose uptake and utilization** | **1.72**±**0.5** | **2.49**±**1.04** |  |
| Fucose permease | 0.17±0.08 | 0.29±0.14 |  |
| Possible fucose ABC transporter (ATP-binding, permease, substrate binding components) | 0.56±0.05 | 0.36 ±0.25 |  |
| L-fucose isomerase | 0.67±0.31 | 0.98±0.36 |  |
| L-fuculokinase | 0.21±0.05 | 0.43±0.27 |  |
| L-fuculose phosphate aldolase | 0.05±0.02 | 0.18±0.15 |  |
| L-fucose mutarotase | 0.05±0.03 | 0.10±0.12 |  |
| **Galactose and Lactose uptake and utilization** | **5.47**±**1.30** | **2.94**±**1.06** |  |
| Lactose phosphotransferase system repressor | 0.10±0.05 | 0.08±0.03 |  |
| Lactose permease | 0.02±0.04 | 0.03±0.03 |  |
| Galactose/methyl galactoside ABC transport system (ATP-binding protein MglA, D-galactose-binding periplasmic protein MglB, permease protein MglC) | 3.86±1.31 | 1.20±0.55 | YES (p<0.05) |
| Tagatose 1,6-diphosphate aldolase | 0.03±0.04 | 0.09±0.03 |  |
| Galactokinase | 1.02±0.19 | 1.09±0.45 |  |
| Galactose-1-phosphate uridylyltransferase | 0.38±0.15 | 0.38±0.17 |  |
| **N-acetylglucosamine uptake and utilization** | **5.17**±**0.56** | **5.51**±**1.31** |  |
| N-Acetyl-D-glucosamine ABC transport system (sugar binding protein, permease protein 1&2) | 2.53±0.35 | 2.07±1.54 |  |
| N-Acetyl-D-glucosamine permease | 0.04±0.03 | 0.04±0.04 |  |
| N-acetylglucosamine-6-phosphate deacetylase | 0.68±0.05 | 0.54±0.23 |  |
| Glucosamine-6-phosphate deaminase | 0.93±0.15 | 1.99±0.60 | YES (p<0.05) |
| N-acetylhexosamine 1-kinase | 0.48±0.14 | 0.42±0.11 |  |
| Predicted transcriptional regulator of N-Acetylglucosamine utilization, GntR family | 0.02±0.03 | 0.06±0.02 |  |
| Chitinase | 0.10±0.03 | 0.14±0.16 |  |
| Chitin binding protein | 0.26±0.04 | 0.07±0.07 | YES (p<0.05) |
| **N-acetylgalactosamine uptake and utilization** | **0.12±0.02** | **0.12**±**0.12** |  |
| N-acetylgalactosamine-specific PTS system components IIB and IID | 0.05±0.02 | 0.01±0.02 | YES (p<0.05) |
| galactosamine-specific PTS system components IIB,IIC and IID | 0.02±0.01 | 0.0±0.01 |  |
| GALNS arylsulfatase regulator (Fe-S oxidoreductase) | 0.03±0.03 | 0.06±0.08 |  |

**References**

Duck LW, Walter MR, Novak J, Kelly D, Tomasi M, Cong Y, *et al.* (2007). Isolation of flagellated bacteria implicated in Crohn’s Disease. Inflamm Bowel Dis 13:1191-1201.

Jamieson AM, Pasman L, Yu S, Gamradt P, Homer RJ, Decker T, *et al*. (2013) Role of tissue protection in lethal respiratory viral-bacterial coinfection. Sci 7:1230-1234.

Kernbauer E, Maier V, Stoiber D, Strobl B, Schneckenleithner C, Sexl V, *et al.* (2012). Conditional Stat1 ablation reveals the importance of interferon signaling for immunity to *Listeria monocytogenes* infection. PLoS Pathog 8:e1002763.

Louis P, Flint HJ. (2007). Development of a semiquantitative degenerate real-time PCR-based assay for estimation of numbers of butyryl-coenzyme A (CoA) CoA transferase genes in complex bacterial samples. Appl Environ Microbiol 73:2009–2012.

Magoč T, Salzberg SL. (2011). FLASH: fast length adjustment of short reads to improve genome assemblies. Bioinformatics 27:2957-2963.

Putz EM, Prchal-Murphy M, Simma OA, Forster F, Koenig X, *et al.* (2012). PI3Kδ is essential for tumor clearance mediated by cytotoxic T lymphocytes. PLoS ONE 7:e40852.

Shang T, Zhang X, Wang T, Sun B, Deng T, Han D. (2011). Toll-like receptor-initiated testicular innate immune responses in mouse Leydig cells. Endocrinol 152:2827-2836.

Urich T, Lanzén A, Qi J, Huson DH, Schleper C, Schuster SC. (2008). Simultaneous assessment of soil microbial community structure and function through analysis of the meta-transcriptome. PLoS ONE 3:e2527.

Ydens E, Cauwels A, Asselbergh B, Goethals S, Peeraer L, Lornet G, *et al.* (2012). Acute injury in the peripheral nervous system triggers an alternative macrophase response. J Neuroinflamm 9:176-193.
